# CDK5RAP3, a Novel Nucleoplasmic Shuttle, Deeply Regulates HSF1-Mediated Heat Stress Response and Protects Mammary Epithelial Cells from Heat Injury

**DOI:** 10.3390/ijms21218400

**Published:** 2020-11-09

**Authors:** Yangyang Shen, Yan Zou, Jun Li, Fanghui Chen, Honglin Li, Yafei Cai

**Affiliations:** 1 College of Animal Science and Technology, Nanjing Agricultural University, Nanjing 210095, China; 2019205005@njau.edu.cn (Y.S.); 2018105002@njau.edu.cn (Y.Z.); 2017205005@njau.edu.cn (F.C.); 2 College of Life Sciences, Anhui Normal University, Wuhu 241000, China; lijunplant@163.com; 3 Department of Biochemistry and Molecular Biology, Medical College of Georgia, Augusta University, Augusta, GA 30912, USA; HLI@Augusta.edu

**Keywords:** CDK5RAP3, HSF1, HSP90, nucleoplasmic shuttle, heat stress response

## Abstract

CDK5RAP3 was regarded as the most significant regulator of cellular responses against heat stress, which is associated with dysfunctions of the immune system and animal susceptibility to disease. Despite this, little known about how CDK5RAP3 regulates heat stress response. In this study, CDK5RAP3 conditional Knockout (CKO) mice, CDK5RAP3^-/-^ mouse embryo fibroblasts (MEFs) and bovine mammary epithelial cells (BMECs) were used as an in vitro and in vivo model, respectively to reveal the role of CDK5RAP3 in regulating the heat stress response. The deletion of CDK5RAP3 unexpectedly caused animal lethality after 1.5-h heat stimulations. Furthermore, BMECs were re-cultured for eight hours after heat stress and was found that the expression of CDK5RAP3 and HSPs showed a similar fluctuating pattern of increase (0–2, 4–6 h) and decrease (2–4, 6–8 h). In addition to the remarkably enhanced expression of heat shock protein, apoptosis rate and endoplasmic reticulum stress, the deletion of CDK5RAP3 also affected nucleoplasmic translocation and trimer formation of heat shock factor 1 (HSF1). These programs were further confirmed in the mammary gland of CDK5RAP3 CKO mice and CDK5RAP3^-/-^ MEFs as well. Interestingly, genetic silencing of HSF1 downregulated CDK5RAP3 expression in BMECs. Immunostaining and immunoprecipitation studies suggested a physical interaction between CDK5RAP3 and HSF1 being co-localized in the cytoplasm and nucleus. Besides, CDK5RAP3 also interacted with HSP90, suggesting an operative machinery at both transcriptional level and protein functionality of HSP90 per se. Together, our findings suggested that CDK5RAP3 works like a novel nucleoplasmic shuttle or molecular chaperone, deeply participating in HSF1-mediated heat stress response and protecting cells from heat injury.

## 1. Introduction

Heat stress (HS) can be simply defined as a state in which heat generated by the body, or the heat absorbed from the outside is not sufficiently dissipated to maintain body temperature [1,2]. Under the temperature stress environment, failure to sustain normal physiological response is at the center of heat stress, thereby leading to a series of complex physiological and psychological changes are affected [3,4]. Whether it is a human or an animal, its physical function will be affected by heat stress [5,6]. Primarily, heat stress has direct or indirect effects on human’s mental health indicators such as emotional experience, depression and other emotional disorders, schizophrenia, Alzheimer’s and other organic mental disorders, substance abuse and dependence, suicidal ideation and behavior. Besides, heat stress will also have a great impact on human physiology [7]. In a high temperature environment, human body is under the state of heat stress that sympathetic nerves are excited, a lot of sweating, blood viscosity increases, and the cardiovascular system is high. Under load conditions, the digestive system function is weakened, which causes the original diseases to be aggravated. Severe changes in blood pressure in the early and late stages of heating can induce heart disease, high blood pressure, and coronary heart disease. In addition, the increase in blood viscosity can lead to thrombosis and stroke; the weakened digestive system causes the negative nutritional status of the human body and further aggravates the original organic disease. Animal husbandry is an important pillar of the current world economy. Plus, with global warming, the loss of heat stress to the world economy in animal husbandry is self-evident. For instance, the current world dairy industry accounts for 20% of the total agricultural output value, and many studies have shown that heat stress can cause significant reductions in dairy cow production. It is therefore of primary importance to understand the mechanisms in heat stress to be able to treat the disease at an early stage before other organs are affected.

Heat shock response (HSR) is another central protective mechanism in cells [8], and also a physiological process where organisms and cells respond to various external stress conditions by activating heat shock transcription factors (HSFs) and regulating the expression of heat shock proteins to maintain cell homeostasis [9,10]. Heat shock factors (HSFs) are transcriptional regulators of genes encoding heat shock proteins as well as numerous other genes associated with cellular processes including development and growth [11]. HSF1 is part of a subfamily which has four heat shock transcription factors, HSF1, HSF2, HSF3, and HSF4, and HSF1 is the major transcription factor for the heat shock response. The induction of HSR requires the activation and translocation to the nucleus of heat shock transcription factors (HSFs) which controls the expression of heat shock proteins (HSPs) [12,13,14,15]. When cells face a stress environment such as elevated temperature, increased heavy metal content, oxidation, etc., external signals transform HSF1 present in the cell to be converted from a monomeric form without DNA binding activity to a trimeric form with DNA binding activity and transfer it to the nucleus, where it binds to the corresponding promoter to initiate the transcription process of the gene and ultimately promote the expression of HSPs [16,17]. 

Yang et al. found that depletion of CDK5RAP3 triggered endoplasmic reticulum stress and activated unfolded protein responses in hepatocytes [18]. Jiang et al. reported the involvement of Cdk5 activator p35-binding protein C53 in the regulation of apoptosis induced by genotoxic stress through modulating the Cdk1-cyclin B1 function [19]. Previous studies showed that CDK5RAP3 is an interesting regulator of the cellular stress response, but it has not been determined whether CDK5RAP3 can protect mammary epithelial cells from heat stress or regulate HSPs transcription via HSF1.CDK5RAP3 is a cyclin-dependent kinase 5 regulatory subunits associated protein 3 [18,19,20,21]. It was first identified as a binding protein of CDK5 (cyclin-dependent kinase 5) activator protein P35 and P39 by yeast two-hybrid technique. CDK5RAP3 activates the tumor suppressor p53 and inhibits growth of tumor cell lines in vitro [22], and it (also known as Cdk5rap3 and LZAP) potentiates DNA damage-induced cell death by modulating the G2/M checkpoint [23,24,25]. CDK5RAP3 is a novel negative regulator of checkpoint response. By counteracting Chk1, which can promote Cdk1 activation and mitotic entry in both unperturbed cell-cycle progression and DNA damage response [26]. In recent years, there have been a large number of studies focusing on the role of CDK5RAP3 in cancer and other aspects [18,27,28,29,30,31,32]. Wang, J.L. et al. found that CDK5RAP3 plays a significant role in NF-kB regulation and tumor suppression. Depletion of CDK5RAP3 increased cellular invasion and MMP-9 expression both of which were dependent on NF-kB and associated with in vitro cellular transformation. Meanwhile, in vivo studies revealed that diminished CDK5RAP3 levels enhanced xenograft tumor growth and angiogenesis. Loss of CDK5RAP3 protein was observed in approximately 30% of human head and neck squamous cell carcinomas (HNSCC), and in these tumors, CDK5RAP3 expression inversely correlated with expression of a subset of NF-kB target genes [33].

However, there are few reports on the mechanism of CDK5RAP3 regulating heat stress responses up till now. In our study, CDK5RAP3 transgenic mouse model, CDK5RAP3^-/-^ MEFs and BMECs were first used to investigate how CDK5RAP3 worked in heat stress response. The reason of choosing BMECs and mammalian gland as the model in vivo and vitro was that they were hypersensitive to thermal stimulation [34,35,36]. Our study suggested CDK5RAP3 can be used as a promising target and protect mammary epithelial cells from the damage induced by heat stress.

## 2. Results

### 2.1. CDK5RAP3 and HSPs Showed Similar Expression Trend in BMECs after Exposure to Heat Stress

**Figure 1:**
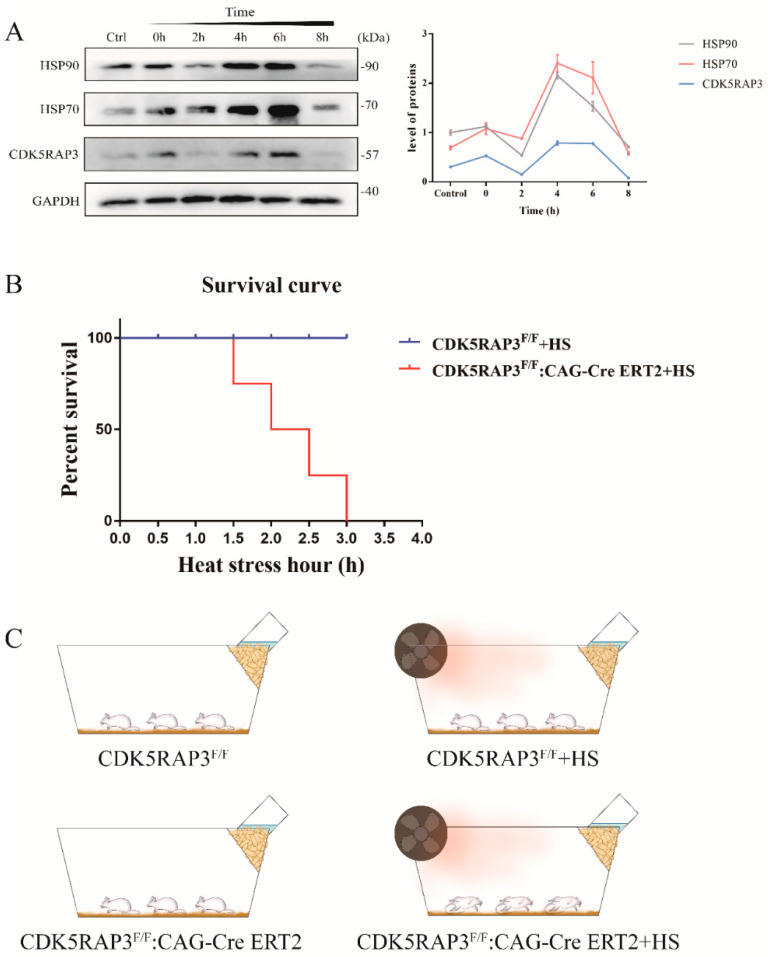
(**A**). CDK5RAP3 and HSPs showed similar expression trends in bovine mammary epithelial cells after exposure to heat stress. The bovine mammary epithelial cells were heat-stressed at 42 °C for 3 h, then they were put back into the cell incubator for recovery culture, and cell proteins were collected at the time points of 0 h, 2 h, 4 h, 6 h and 8 h respectively for western blot analysis. (**B**). The survival curve of CDK5RAP3^F/F^ and CDK5RAP3^F/F^: CAG-Cre ERT2 mice after heat stress. The abscissa is the time of heat stress. (**C**). Experimental model of mice subjected to heat stress. All the experimental mice were in the lactation period, the method of obtaining CDK5RAP3^F/F^: CAG-Cre ERT2 mice were detailed in the Section 4.

To investigate the effect of CDK5RAP3 on mammary epithelial cells induced by heat stress, BMECs were subjected to heat stimulation at 42 °C. HSP90 and HSP70 increased significantly after three hours of heat stress, then decreased markedly after two hours’ recovery culture. Curiously, the expression showed a sharp rise at 4 h, and gradually dropped to the control protein expression levels at 6 and 8 h (Figure 1A). 

### 2.2. The Deletion of the CDK5RAP3 Gene Accelerated the Death of Mice

In order to observe whether the deletion of the CDK5RAP3 gene has an effect on the survival of mice under heat stress of 42 degrees, an experiment was conducted with CDK5RAP3^F/F^; CAG-Cre ERT2 conditional knockout mice and wild-type mice at lactation stage (Figure 1C). The results showed that the whole body knockout CDK5RAP3 mice died within 1.5 h under 42 °C heat stress, while wild-type mice survived for more than three hours (Figure 1B). It was also found that CDK5RAP3 deletion would not cause animal death. Interestingly, when CKO mice were subjected to heat stress at 42 °C, their sudden death at 1.5 h made us curious about how CDK5RAP3 gene played a role in the heat shock response. 

### 2.3. CDK5RAP3 Induces Apoptosis Due to the Heat Stress

**Figure 2:**
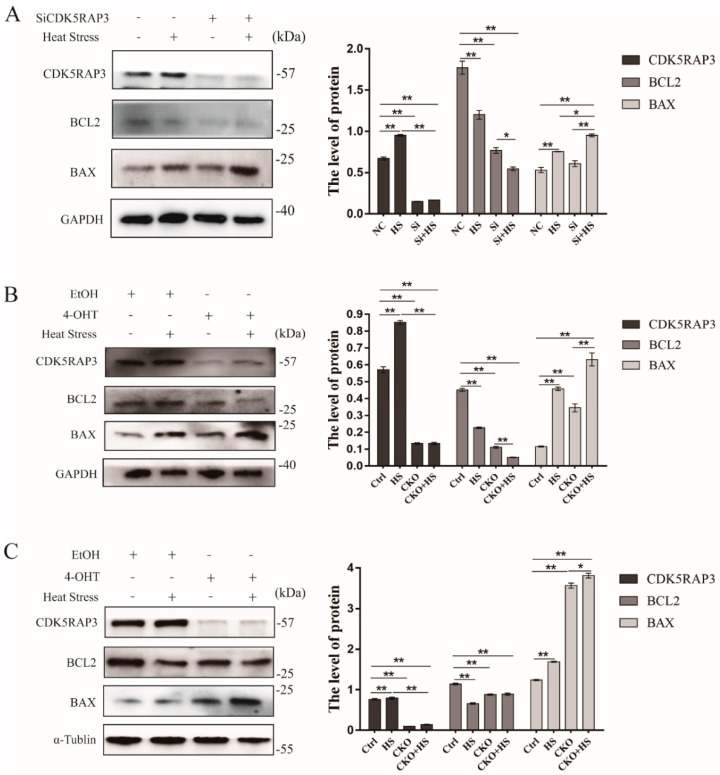
CDK5RAP3 induces apoptosis in bovine mammary epithelial cells, mouse fibroblasts and mammary gland of mice due to heat stress. Expression of apoptosis marker genes of protein BAX, BCL2 in bovine mammary epithelial cells (**A**), mouse fibroblasts (**B**) and mammary gland of mice (**C**) cultured under heat stress (42 °C 3 h) and corresponding control (37 °C). The BMECs were silenced by using the SiRNA of CDK5RAP3, marked as ‘+’. Cre-ERT2 mice are a type of mice that express the fusion protein of estrogen receptor (ER) ligand binding region mutant (ERT) and Cre recombinase. Cre-ERT2 is inactive in the cytoplasm without the induction of Tamoxifen; when induced by Tamoxifen, 4-OHT (estrogen analogue), which is a metabolite of Tamoxifen, and would bind to ERT, allowing Cre-ERT2 to enter the nucleus and exert Cre Recombinase activity. Tamoxifen (4-OHT) were injected to knock out the CDK5RAP3 gene in mouse fibroblasts cells, marked as “+”. The method of obtaining and CDK5RAP3^F/F^: CAG-Cre ERT2 mice were detailed in the Materials and Methods. Alcohol (EtOH) was the negative control. Meanwhile, those not treated accordingly were marked as ‘-’. GAPDH and α-Tublin were used to normalize the expression of targets protein expression of BAX, BCL2. Those results are expressed as the mean ± SEM. of *n* = 3, * *p* < 0.05, ** *p* < 0.001 compared with the control.

In order to evaluate the effect of CDK5RAP3 on the heat stress-induced apoptosis, the expression of apoptotic genes such as BAX and BCL2 was studied in cells and mice. Ours results indicated that heat stress induces the upregulation of BAX genes in MEFs, mammary gland and BMECs. What’s more, elevation of BAX gene was enhanced when CDK5RAP3 gene was knockdown and exposed to heat stress in vivo or vitro. On the contrary, heat stress induces the down-regulation of BCL2 genes and a modest reduction in protein expression of BCL2 gene when CDK5RAP3 was knockdown. (Figure 2A–C). These results suggested that CDK5RAP3 is involved in cell death program, and the absence of CDK5RAP3 promotes the heat stress-induced cell death. 

### 2.4. Regulation of the Heat Shock Factor 1-Mediated Stress Response Pathway by CDK5RAP3

#### 2.4.1. CDK5RAP3 Depletion Inhibited the Expression of HSP90, While HSP70 Showed No Significant Change

**Figure 3:**
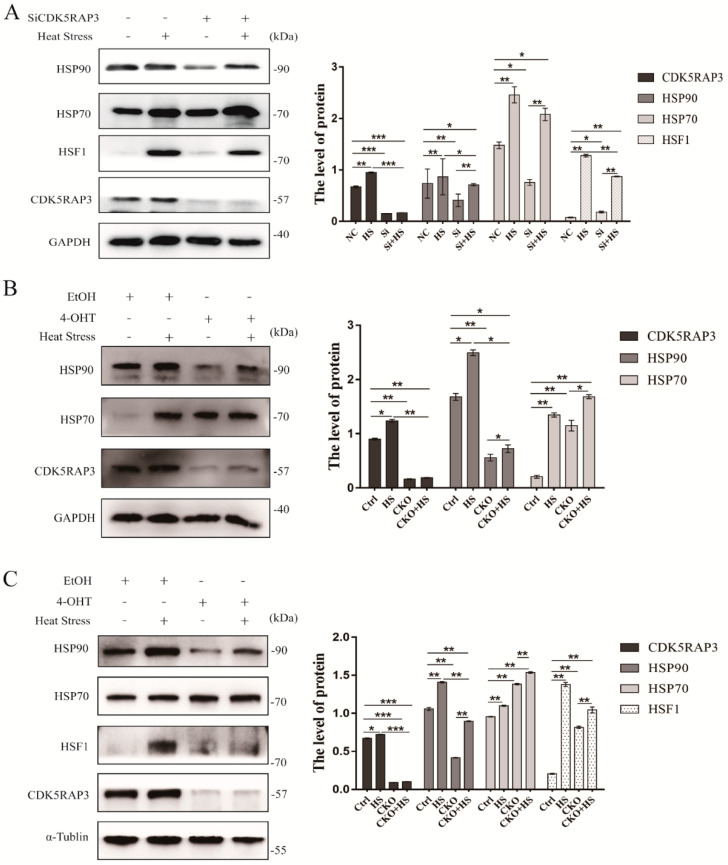
CDK5RAP3 deletion inhibited the expression of HSP90, while HSP70 showed no significant change. Protein accumulation of HSP70 and HSP90 in bovine mammary epithelial cells (**A**), mouse fibroblasts (**B**) and mammary gland of mice (**C**) cultured under heat stress (42 °C 3 h) and corresponding control (37 °C). The BMECs were silenced by using the SiRNA of CDK5RAP3, marked as ‘+’. Tamoxifen(4-OHT) were injected to knock out the CDK5RAP3 gene in mouse fibroblasts cells, marked as ‘+’. The method of obtaining and CDK5RAP3^F/F^: CAG-Cre ERT2 mice were detailed in the Materials and Methods. Alcohol (EtOH) was the negative control. Meanwhile, those not treated accordingly were marked as ‘-’. GAPDH and α-Tublin were used to normalize the expression of targets protein expression of HSP90, HSP70 and HSF1. Those results are expressed as the mean ± SEM. of *n* = 3, * *p* < 0.05, ** *p* < 0.01, *** *p* < 0.001 compared with the control.

Heat shock response is a physiological process of maintaining intracellular homeostasis by activating heat shock transcription factors (HSFs) and up-regulating the expression of heat shock proteins (HSPs). To further investigate the level of heat shock response induced by CDK5RAP3 deficiency under heat stress, an experiment was performed on the MEFs, mammary gland and BMECs. HSP90 and HSP70 in the three membrane types was found increased after heat stress at 42 °C, indicating that the heat stress response was activated. However, after the knock down of CDK5RAP3, the expression of HSP90 was sharply dropped, while there was no significant difference in HSP70 (Figure 3A–C). In brief, both in vitro and in vivo results indicated that CDK5RAP3 was involved in heat stress response and affected the expression of HSP90. 

#### 2.4.2. The Knockdown of CDK5RAP3 Did Not Affect the mRNA Expression Level of HSF1, But the Expression of HSF1 Protein

**Figure 4:**
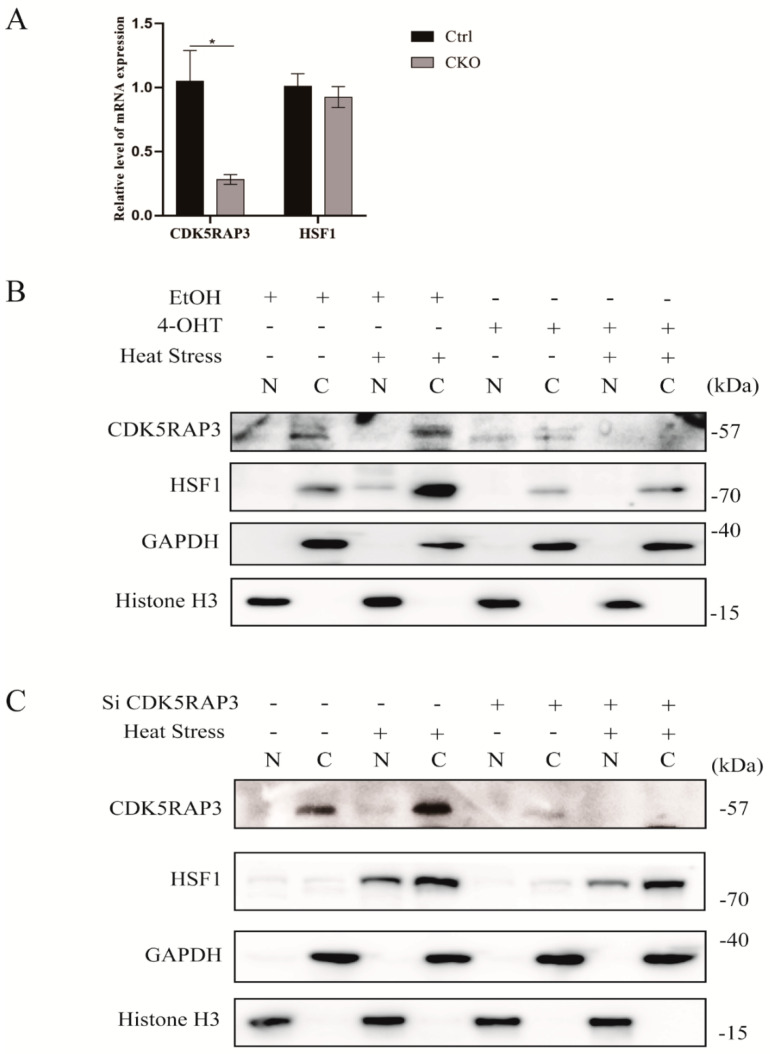
(**A**). The knockdown of CDK5RAP3 did not affect the mRNA expression level of HSF1, but the protein expression of HSF1. mRNA expression of HSF1(A) in mouse fibroblasts. The results are expressed as the mean ± SEM. of *n* = 3, * *p* < 0.05 compared with the control. (**B**,**C**). The knockout of CDK5RAP3 gene affected the nuclear and cytoplasmic distribution of HSF1. Nuclear and cytoplasmic separation experiment. CDK5RAP3mefs-control or BMECs-NC and CDK5RAP3Mefs-CKO or BMECs-SiCDK5RAP3 cells were divided into two dishes respectively, one dish was used as the control group, the other was stimulated by heat at 42 °C for 3 h as the HS group. Trypsin digested and collected the cell precipitates. C for the cytoplasm, N for the nucleus. GAPDH was cytoplasmic protein reference and Histone H3 was nuclear protein reference.

HSF1 is a heat shock transcription factor that plays a leading role in heat shock response. Our previous results showed that the protein level of HSP70 did not change notably after the knock down of CDK5RAP3 under heat stress at 42 °C, while the protein expressions of HSP90 and HSF1 were both reduced. To investigate whether the decrease of HSP90 protein level was on account of the decrease of HSF1 mRNA, the level of HSF1mRNA was tested in MEFs. qRT-PCR results showed that there was no significant difference in the mRNA expression level of HSF1 when CDK5RAP3 gene was knocked down, suggesting that the CDK5RAP3 gene knocked down had no influence on the transcription level of HSF1 (Figure 4A). Meanwhile, we checked the effect of CDK5RAP3 deletion of HSF1 in BMECs (Figure 3A) and mammary gland on the protein level (Figure 3C). Both in vitro and in vivo results showed that HSF1 was expressed fairly low under normal conditions, also consistent with previous reports. When exposed to heat stress, the expression of HSF1 increased greatly. While the expression of HSF1 was decreased after CDK5RAP3 was knockdown. To summarize, CDK5RAP3 did not regulate the transcription of HSF1 mRNA but affected the expression of HSP90 via affecting HSF1 protein level.

#### 2.4.3. The Knockout of CDK5RAP3 Gene Affected the Nuclear and Cytoplasmic Distribution of HSF1 and Inhibited the Trimer Formation of HSF1

Previous experimental results showed that CDK5RAP3 knockout affected the expression of HSF1 and HSP90. In the normal physiological state of cells, HSF1 is located in the cytoplasm as a monomer. When cells are stimulated by the external environment, HSF1 can only play its role in regulating the transcription of heat shock proteins from the cytoplasm to the nucleus. To figure out whether the differentiation of protein expression between HSF1 and HSP90 was caused by the different nuclear and cytoplasmic distribution of HSF1, nuclear and cytoplasmic separation experiments was conducted on BMECs, MEFs under heat stress and without external environmental stimulations. When cells were subjected to heat stress at 42 °C, HSF1 expression increased and phosphorylation occurred, most of them entered the nucleus from the cytoplasm. However, after CDK5RAP3 gene knockout cells stimulated by heat, the expression of HSF1 decreased, and most of them were transferred from the nucleus into the cytoplasm (Figure 4B,C). This results indicated that CDK5RAP3 knockout affected the nuclear and cytoplasmic distribution of HSF1.

**Figure 5:**
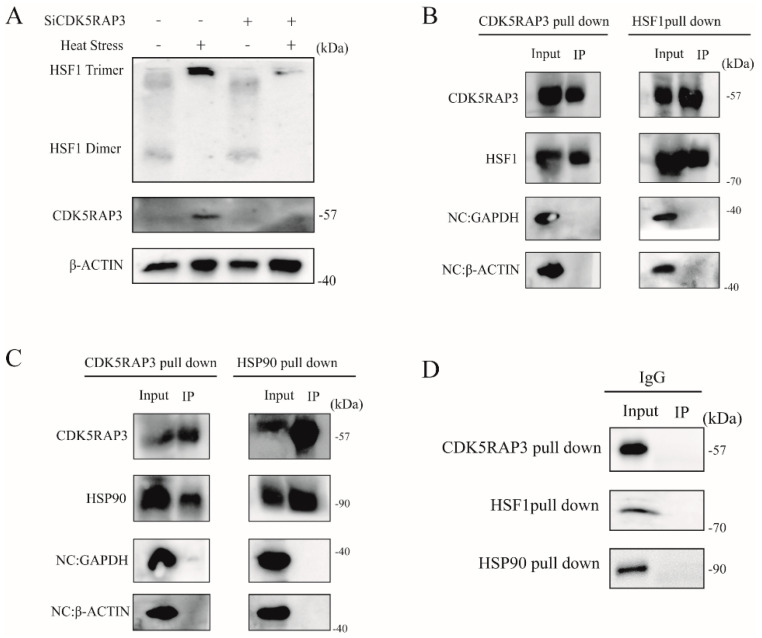
(**A**). The knockout of the CDK5RAP3 gene inhibited the trimer formation of HSF1. The BMECs were silenced by using the SiRNA of CDK5RAP3, marked as “+”. BMECs-NC and BMECs-SiCDK5RAP3 cells were divided into 2 dishes respectively, and the cells were treated in two ways: one dish remained untreated (control group), while the other was heat-stressed at 42 °C for 3 h (HS group). The cells were lysed with IP lysate and cross-linked protein lysate with a final concentration of 0.12% glutaraldehyde at room temperature for 5 min, followed by 1M Tris-HCl (PH 8.0) to terminate the crosslinking. Due to the large molecular weight of the protein after crosslinking, 8% separation gel was used for electrophoresis. beta-actin is the internal reference. (**B**–**D**). CDK5RAP3 interacts with HSF1 to influence the expression of HSP90. Endogenous CDK5RAP3 was immunoprecipitated with CDK5RAP3 polyclonal antibody, and the presence of HSF1 and HSP90 was detected with corresponding antibodies.

When HSF1 enters the nucleus as a monomer, it has no transcriptional activity, and HSF1 must form homologous trimers to exert its transcriptional activity. Therefore, whether the deletion of CDK5RAP3 would affect the degree of the trimer formation of HSF1 in the nucleus was further suspected. To confirm this conjecture, the trimer formation state of HSF1 (Figure 5A) was examined. And the experimental results showed that most HSF1 exists in the form of the dimer, while a small part of HSF1 exists in the form of the trimer. When heated at 42 °C, HSF1 basically exists in the form of the trimer. By contrast, after CDK5RAP3 was knocked out, the trimer of HSF1 was significantly reduced during heat stress, suggesting that CDK5RAP3 knockout could inhibit the trimer formation of HSF1 and thus affect the activation level of HSF1 and HSP90.

#### 2.4.4. CDK5RAP3 Interacts with HSF1 to Influence the Expression of HSP90

**Figure 6:**
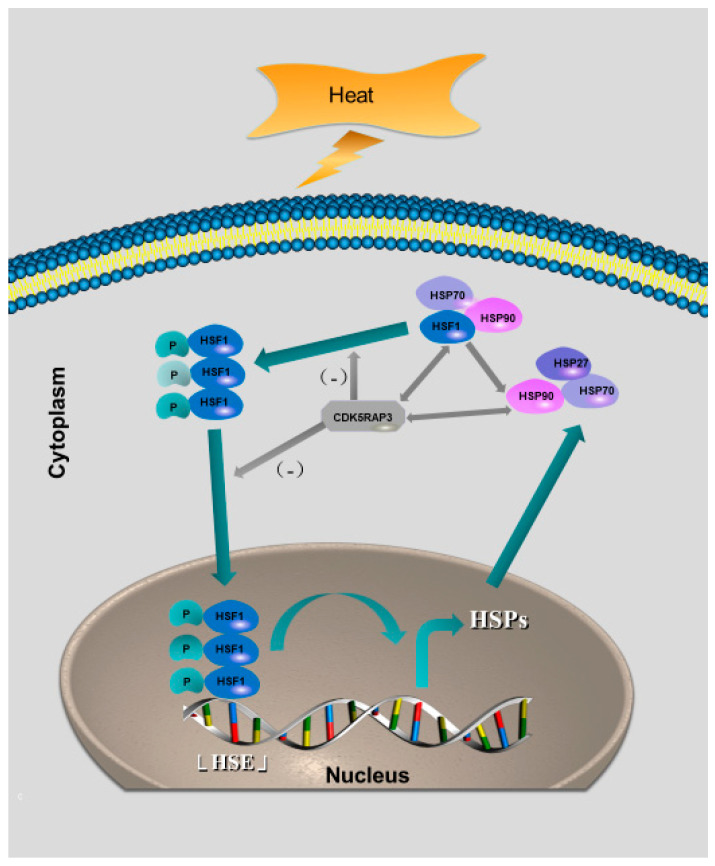
A preliminary illustration of the heat stress response mechanism mediated by CDK5RAP3. Normally, HSF1 is located in the cytoplasm and binds to the heat shock protein in an inactivated state. When the cells are stimulated by the external environment, such as high temperature, HSF1 will be activated and transformed from a monomer into a trimer. CDK5RAP3 can inhibit the activation of HSF1, inhibit the formation of HSF1 trimer, and affect the nuclear and cytoplasmic distribution of HSF1, thus participating in the heat shock response and affecting the expression of heat shock protein. However, whether CDK5RAP3 affects the formation of HSE has yet to be verified.

Our previous results showed that CDK5RAP3 did not affect the transcription of HSF1mRNA, but could regulate the expression of HSP90 by influencing the protein expression, the nuclear and cytoplasmic distribution, and the trimer formation of HSF1. Therefore, we further suspected whether CDK5RAP3 would interact with HSF1 to affect the expression of HSP90.Futhermore, to demonstrate our initial observation of the interactions among CDK5RAP3 and HSF1, co-immunoprecipitation (co-IP) assays using bovine mammary gland was first performed. (Figure 5B–D) As shown in Figure 5B,C, HSF1 and HSP90 were both present in the CDK5RAP3 pulldown immunoprecipitations, and CDK5RAP3 was present in the HSF1 and HSP90 pulldown immunoprecipitations as well. Endogenous HSF1 and HSP90 were co-immunoprecipitated with endogenous CDK5RAP3 in mammary gland, indicating that CDK5RAP3 may not only interact with HSF1 but also i HSP90 directly to jointly affect whose expression (Figure 6). However, how CDK5RAP3 specifically interacts with HSF1 and HSP90 is a complex process that needs further evidence to be proved.

#### 2.4.5. The Knockdown of HSF1 Affect the mRNA and Protein Expression Level of CDK5RAP3 in BMECs

**Figure 7:**
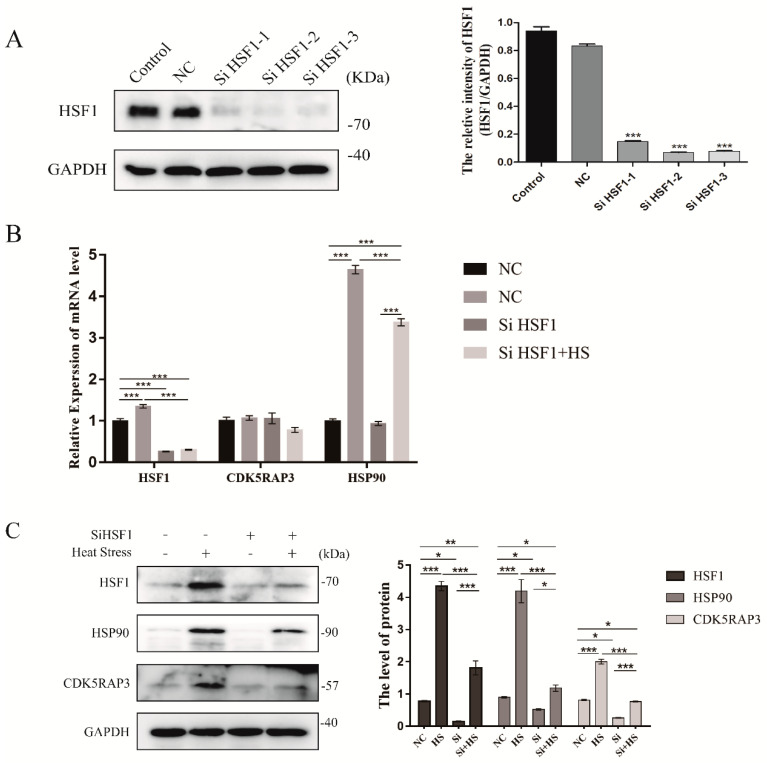
(**A**). The transfection efficiency of bovine HSF1 siRNA was detected by Western blot. (**B**,**C**). The knockdown of HSF1 affected the mRNA and protein expression level of CDK5RAP3 in BMECs. mRNA expression of HSF1(B), protein accumulation of HSF1(C) in BMECs cultured under heat stress (42 °C 3 h) and corresponding control (37 °C). The BMECs were silenced by using the SiRNA of HSF1, marked as “+”. GAPDH was used to normalize the expression of targets protein expression of HSF1. The results are expressed as the mean ± SEM. of *n* = 3, * *p* < 0.05, ** *p* < 0.01, *** *p* < 0.001 compared with the control.

The results of Co-IP showed that CDK5RAP3 would interact with HSF1, affecting the expression of HSP90. We have previously shown that CDK5RAP3 deletion influenced the expression of HSF1 protein, so we speculated whether the expression of CDK5RAP3 mRNA and protein was regulated by HSF1. Therefore, the HSF1 gene was silenced in cells to detect the two indexes. Real-time PCR and western blot showed that the mRNA and protein expression levels of CDK5RAP3 were remarkably decreased after HSF1 gene knockout, indicating that HSF1 gene knockout had an impact on the transcription and protein levels of CDK5RAP3 (Figure 7A–C).

#### 2.4.6. Immunofluorescence Results Show that CDK5RAP3 Binds to HSF1 

**Figure 8:**
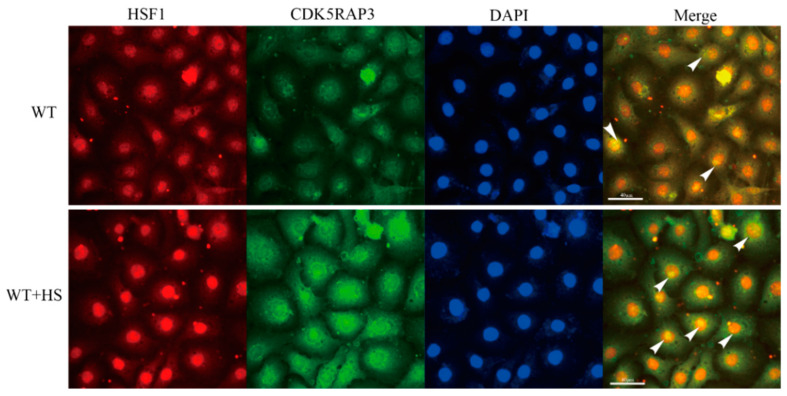
DAPI is the staining of the nucleus, and the nucleus is blue light. The CDK5RAP3 shows a green light. HSF1 shows a red light. The yellow light indicates that there is a binding between CDK5RAP3 and HSF1, and the binding site is indicated by a white arrow. Merge is a synthesis diagram of the cytoplasm and nucleus. The scale bart “40 μm” is indicated by a white straight line in Merge.

Previous studies have shown that HSF1 is abundant in the cytoplasm under normal circumstances and is inactive when combined with heat shock proteins. When stimulated by the external environment, HSF1 enters the nucleus and works, while CDK5RAP3 is located in the cytoplasm, CDK5RAP3 plays its part in the homeostasis regulation of cells under stress. There were few reports focused on whether it will enter the nucleus to play a role. Interestingly, our pre-existing results showed that CDK5RAP3 interacts with HSF1 and HSP90. At the same time, the expression of CDK5RAP3 mRNA and protein is also regulated by HSF1. Similarly, the protein expression of HSF1 is affected by CDK5RAP3. Hence, we speculate whether CDK5RAP3 will enter the nucleus, bind to HSF1, and work together to affect the expression of HSP90. And, immunofluorescence co-staining technology was used to observe CDK5RAP3 and HSF1 in BMECs (Figure 8). The results revealed that CDK5RAP3 and HSF1 are bound in the cytoplasm with a small amount under normal conditions. Obviously, there is a union between them, which is consistent with our conjecture.

### 2.5. Alteration in Expression of GRP78 and CHOP Due to Deficiency of CDK5RAP3 and Heat Stress

**Figure 9:**
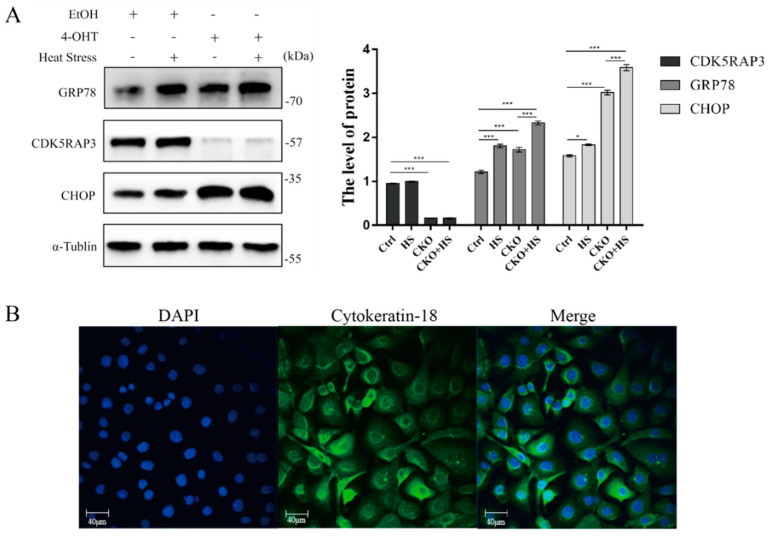
(**A**). Alteration in expression of GRP78 and CHOP due to deficiency of CDK5RAP3 and heat stress. Expression of ER stress marker genes protein accumulation of GRP78 and Chop in mammary gland of mice cultured under heat stress (42 °C/3 h) and corresponding the control (37 °C). The BMECs were silenced by using the SiRNA of CDK5RAP3, marked as “+”. α-Tublin was used to normalize the expression of targets protein expression of GRP78 and Chop. The results are expressed as the mean ± SEM. of *n* = 3, * *p* < 0.05, *** *p* < 0.001 compared with the control. (**B**). Identification of bovine mammary epithelial cells by immunofluorescence. DAPI is the staining of the nucleus, and the nucleus is blue light. Cytokeratin-18 is the localization of immunofluorescence expression of keratin-18, a protein specific to epithelial cells. Merge is a synthesis diagram of the cytoplasm and nucleus.

To determine the potential crosstalk among CDK5RAP3, heat stress and ER stress response in mammary gland, GRP78 and CHOP level was investigated in mammary gland exposed to 42 °C for 3 h compared to those cultured at 37 °C (Figure 9A) Accordingly, compared to the control group, the expression of GRP78 and CHOP genes notably increased in the CDK5RAP3 CKO group under 42 °C 3 h, while there was no big difference between CDK5RAP3 knockout heat treatment (CKO+HS) and CDK5RAP3 knockout no heat treatment (CKO). Interestingly, GRP78 and CHOP protein level were both higher in the group of CDK5RAP3 knockout than the wild-type group. These solid evidences confirmed the crosstalk among them.

## 3. Discussion

Herein we have delineated the molecular mechanisms of CDK5RAP3 in heat-stressed mammary glands through HSF1. In recent years, there have been more and more studies focusing on CDK5RAP3 in mammals. Many previous works have determined the abnormal expression of CDK5RAP3 in different heat-stressed tissues or cell lines, also its important regulatory effects on different diseases, while few researches focused on the molecular mechanism of heat stress.

We have shown that the expression of CDK5RAP3 and HSPs showed a similar fluctuating pattern. This drastic change may be related to the homeostasis of the cellular environment in order to protect itself from heat stress. Interestingly, the expression changes of CDK5RAP3 protein were almost identical to those of HSP90 and HSP70. However, the expression of CDK5RAP3 protein was significantly reduced after two hours of recovery compared to heat shock protein. This also suggested that CDK5RAP3 is in response to heat stress factors, which defines much of the heat shock state in these cells, and leading to heat shock response that induces expression of downstream gene under stress stimulation, with the goal of reconstituting the dynamic balance of intracellular protein. 

We demonstrated that CDK5RAP3 plays an anti-apoptotic role in the cellular heat shock response. Under normal physiological conditions, the expression level of heat shock protein is fairly low, accounting for only 1–2% of the total protein. When stimulated by heat shock, excessive reactive oxygen species or inflammation, HSF1 is activated and then binds to the promoter of the downstream heat shock protein gene, promoting the expression of heat shock proteins, thereby maintaining the intracellular homeostasis [37]. Heat shock proteins can be divided into two types according to their molecular weights: one is ATP-dependent Macromolecular HSPs, such as HSP100, HSP90, HSP70 and HSP60; the other is ATP-independent macromolecular HSPs, such as HSP25 and αB-crystalline [13,38,39,40,41]. 

Importantly, with the knockdown of CDK5RAP3, the trimer of HSF1 revealed in the HSF1 trimerization was significantly reduced. We suspected that due to the deletion of CDK5RAP3, HSP90 has different regulations of HSF1. Under normal conditions, HSP90 and other proteins, such as p23 and cyclophilins (CY), form a protein complex together and bind to HSF1 to inhibit its transcriptional activity. Protein stress can remodel the inhibitory protein complex represented by HSP90, causing a conformational change in HSF1 to activate it. HSP90 is a soluble protein which is normally localized in the cytoplasm [42]. The interaction between HSF1 and HSP90 is a dynamic process [10]. Therefore, the co-immunoprecipitation assay was used to explore the interaction between HSF1 and HSP90 in these two cells to further explore the molecular mechanism of HSF1 activated. (Figure 6) The formation of complex CDK5RAP3-HSF1-HSP90 and its connection with the HSP90 expression was also revealed in IP assays. It is interesting that this compound does not contain HSP70, while both HSP70 and HSP90 bind HSF1 to prevent its activation. However, whether CDK5RAP3 is a competitor of HSP70 in the regulatory interactions with HSF1 and whether the known inhibitors of HSP90 chaperone activity (e.g., 17AAG of AUY922) are able to disrupt the CDK5RAP3-HSF1-HSP90 compound. And other inducible HSPs (HSP27, HSP40, HSP60, others), whose expression is triggered by activated HSF1, whether their expression can be altered via the revealed CDK5RAP3/HSF1 pathway still need to be verified in the future research. This is particularly important considering that little has been known about how to target the function of HSF1 and HSP90 via inhibiting CDK5RAP3. Taking into consideration that both HSF1 and HSP90 are ones of the factors defining tumor growth and tumor resistance to therapeutics, such knowledge is crucial for a rational development that CDK5RAP3 may be examined as a potential target for anticancer therapy.

With regard to the hsp90-based molecular chaperone compound, which has been mentioned above in vitro, other molecular chaperones were involved in regulating the activity of HSF1 as well. For instance, HSBP1 (heat shock factor blinding protein1) inhibits the activation of HSF1 monomer, while DAXX (Fas death domain-associated protein) promote HSF1 activation. DAXX is a transcriptional regulatory protein mainly distributed in the nucleus and was named after its participations in the Fas apoptosis pathway. Yeast two-hybrid system found that DAXX interacts with HSF1 trimer. Functional studies have shown that overexpression of DAXX can strongly enhance the transcriptional activity of HSF1 trimers (does not affect HSF1 monomer); on the contrary, when DAXX is lacking, heat stress cannot induce HSP70 expression. The role of DAXX is related to its ability to competitively block the binding of HSP90-p23-FKBP52 complex to HSF1 trimer. Noted that, in our study, the absence of C53 would affect the formation of HSF1 trimerization was also found. At the same time, no literature has reported whether C53 interacts with DAXX so as to jointly participate in the regulation of HSF1 trimerization. In consequence, we will focus on the relationship between HSF1 and DAXX in the following research.

In order to explore whether the phosphorylation of HSF1 in this study is regulated by CDK5RAP3, thus affecting the transcription activity of HSF1. We examined the phosphorylation of serine at site 326 under the influence of CDK5RAP3. At present, the potentially target band has not been detected by western blot yet, which might be due to the antibody, or the phosphorylation did not occur. HSF1 is largely subjected to various post-translational modifications such as phosphorylation, acetylation, SUMO, and so on under heat stress, which can regulate its DNA binding activity and transcriptional activity [43,44]. Up to now, 22 sites on the serine and threonine residues of the HSF1 protein have been phosphorylated, such as S326, S303, S307 and so on. For example, the phosphorylation of Ser230 and Ser326 is associated with HSF1 activation; while the phosphorylation of Ser121, Ser303, Ser307 and Ser363 is associated with HSF1 inactivation. Among them, Ser-326 phosphorylation on HSF1 was identified as a key modification of HSF1 activation under pressure induction. This modification was originally found to be mediated by the mTOR signal [45]; however, a new study suggested that the RAS/MAPK signaling pathway regulates HSF1 activation by phosphorylating the 326 serine site [46]. Next, we will further detect whether CDK5RAP3 affects phosphorylation of HSF1 at position 326 or other sites of HSF1.

The purpose of this study was to analyze the role of CDK5RAP3 on the heat stress mammary gland and its regulatory mechanism (Figure 6). The herein presented experimental data and the model of CDK5RAP3-deleted mammary gland epithelial cells, CDK5RAP3 mice embryonic fibroblasts and CDK5RAP3 tamoxifen inducible conditional knockout (CAG-Cre ERT2) mice demonstrate that the effects of CDK5RAP3 on heat-stressed mammary glands. The model thus provides a unique tool to study the formation mechanism of heat stress, and provide references for anti-heat stress of CDK5RAP3 at the organ level of human health for the following study and the pathogenesis of heat stress provides a comprehensive theoretical basis for relieving heat stress. As the greenhouse effect intensifies, the heat stress becomes more and more prominent, which not only does it pose a threat to human health, but also has a huge impact on the world economy, such as the dairy industry [47,48]. The typical characteristic of the heat stress response is the expression of heat shock protein. After stimulating cells with high temperature, it adapts to the change in external temperature by activating the cell signaling pathway and inducing the expression of programmed gene. In the past decade, we have learned a lot about the regulation mechanism of heat stress. However, how to develop targeted drugs in the future to alleviate the heat stress is worth further exploration, with this new type of understanding of the heat stress now available in our study, the new approach in practice of CDK5RAP3 should benefit from the unique insights gained from our data and model.

## 4. Materials and Methods

### 4.1. Animals

A total of 30 females and 10 male C57BL/6J mice (6–8 weeks old, weighing 20–25 g) were kept in our SPF mice house. These mice were cohabited with the ratio of 3 (female) to 1 (male) for conception and housed separately after pregnancy until five to seven days after calving. Lactating female mice were raised at 24 ± 1 °C with 40–80% humidity and allowed access to food and water ad libitum. Feeding and management were executed in accordance with the relevant regulations of the National Institutes of Health.

All experimental procedures and protocol were designed and approved in accordance with the guidelines for the animal welfare and use of animals, prepared by the Institutional Animal Care and Ethical Committee of Nanjing Agricultural University (Permit number: SYXK (Su) 2019–0066), Nanjing, China. Complied guidelines for animal care were carefully observed to ensure all animals were treated compassionately and with regard for alleviation of suffering. And all experiments were carried out in strict accordance with the guidelines and rules.

CDK5RAP3 tamoxifen inducible conditional knockout (CAG-Cre ERT2) mice were bred as described in our previous studies [26,49]. Floxed CDK5RAP3 mice were crossed with CAG-Cre ERT2 mice, and CKO mice were generated from self-cross of F1 offspring. The following primers were used for polymerase chain reaction (PCR) genotyping of CDK5RAP3 mice: 

P1: CDK5RAP3^F/F^ HL824 (TAG CTC GGG GCT CAG ACG CTC TGA)

P2: CDK5RAP3 ^F/F^ 825 (TTA TCT GCT CTT CCC GCT AGA ATA)

CDK5RAP3 ^F/F^ PCR condition: 94 °C 4 min, 92 °C 45 s, 55 °C 45 s, 72 °C 45 s, 40 cycles, 72 °C 10 min;

WT(-/-): a lower band, Hetero(+/-): upper and lower bands, Homo(+/+): upper band.

Genotyping of CAG-Cre ERT2 mice were followed by the protocol of Jackson Laboratory. The following primers were used for polymerase chain reaction (PCR) genotyping of CAG-Cre ERT2 mice:

CAG-Cre ERT2 F: (GAA GCA ACT CAT CGA TTG ATT TAC)

CAG-Cre ERT2 R: (CAT CCA ACA AGG CAC TGA CCA TCT)

CAG-Cre ERT2 PCR condition: 94 °C 5 min, 92 °C 30 s, 52 °C 1 min, 72 °C 1 min, 38 cycles, 72 °C 10 min.

CAG-Cre ERT2 (-): no band, CAG-Cre ERT2(+): one band about 480 bp.

### 4.2. Chemical Reagents

Tamoxifen, 4-hydroxytamoxifen and other chemical reagents were purchased from Sigma (Sigma-Aldrich, St. Louis, MO, USA). The whole body knockout CDK5RAP3 was induced by tamoxifen administration. Tamoxifen (stocking buffer: 20 mg/mL in corn oil) was administrated by continuous 5-day intraperitoneal injection with a dose of tamoxifen 75 mg/kg body weight. All animal protocols were approved by the Institutional Animal Care and Use Committees (IACUC) of Nanjing Agricultural University and Augusta University. 

### 4.3. Cell Culture and Heat Treatment

The mouse embryo fibroblasts (MEFs) were prepared using CDK5RAP3^F/F^; CAG-Cre ERT2 embryos at 13 or 14 d.p.c. (day post-coitum). The uterine horn was dissected out, after rinsed with 70% (*v*/*v*) ethanol, they were placed into PBS. Then, the head, limbs and red organs were removed, washed with PBS, the tissues were transferred to 50 mL test tube, and incubated with trypsin at 37 °C for 15 min. After incubation for 5 min, the cells were completely separated by suction up and down, and then approximately 1 volume of freshly prepared MEF medium was added to inactivate trypsin. MEF culture medium (components to make 500 mL of media, mix all components and filter): 450 mL of DMEM, 50 mL of FBS (10% (*v*/*v*)), 5 mL of 200 mM L-glutamine (1/100 (*v*/*v*)), 5 mL of Penicillin-streptomycin (1/100 (*v*/*v*)). Then it was transfected with LT lentivirus. Deletion of CDK5RAP3 and was induced by addition of 4-hydroxytamoxifen (1 mM, Sigma). 4-hydroxytamoxifen was added to the medium at a ratio of 1:7000 and then cells were cultured for 3–4 days.

The MAC-T bovine mammary epithelial cell lines used in this experiment were presented by Professor Yang’s lab of Yangzhou University, China (Figure 9B).

Cells were cultured in DMEM containing 10% FBS at 37 °C in a humidified incubator under 5% CO_2_. The medium was changed for every 2 days. Cells were heat treated for 3 h at 42 °C.

### 4.4. Identification of Bovine Mammary Epithelial Cells by Immunofluorescence

Immunofluorescence steps as follows: after the cells grew to 1 × 10^6^, the medium was aspirated, cells were flushed twice with PBS for 5 min, and fixed with 1 mL 4% paraformaldehyde solution per well for 1 h, washed with PBS 3 times for 5 min each time, then perforated with 0.5% Triton-x100 for 10 min, washed twice with PBS for 5 min, 2% BSA for 1 h, then added diluted keratin 18 with rabbit polyclonal antibody (1:500) to incubate at overnight at 4 °C, washed with 3 times in PBS for 5 min, incubated with anti-rabbit IgG (1:500) for 1 h at room temperature, and flushed 3 times with PBS for 3 times respectively Hoechst 33342 was added to the condense nucleus for 10 min, flushed 3 times with PBS, sealed slices, examined with laser scanning confocal microscope. More than 90% of cells in the field of vision express specific green fluorescence, and cytokeratin-18 staining is positive (Figure 9B).

### 4.5. Total RNA Isolation and qRT-PCR Analysis

Total RNA was prepared from isolated sample by extraction with the RNA Purification Kit (Thermo Scientific, Waltham, MA, USA), and then reversely transcribed using the First-Strand Synthesis System (Invitrogen) following the manufacturer’s instructions. Total RNA concentration and quality were determined using a Nanodrop ultra-micro spectrophotometer. RNA sample from each treatment group was reverse-transcribed to cDNA using a Takara’s reverse transcription kit. The reaction system is as follows: RNA (4 ΜL, 500 ng/μL), 5 × PrimeScript RT Master Mix (4 μL), RNase Free dH2O (12 μL). After the end of the reaction, the obtained cDNA product was adjusted to a concentration and stored at −20 °C for use.

The iTaq Universal SYBR Green Supermix kit (BIO-RAD, Hercules, CA, USA) was used to perform qRT-PCR with 40 cycles of 95 °C for 15 s and 60 °C for 1 min on the StepOnePlusPCR System (LifeTechnologies | Thermo Fisher Scientific - CN, Shanghai, China). The results were analyzed by the StepOne Software (Version 2.1, Life Technologies, Waltham, MA, USA). The targeted gene level was normalized to β-actin and GAPDH by using the 2^−△△CT^ method. The primers used are shown in Table 1. 

### 4.6. The Extraction of Cellular Nuclear and Cytoplasmic Protein

The cells in the 6-well plate were flushed three times with pre-cooled PBS, and the residue was blotted on a blotting paper and placed on ice; 100 μL of protein lysate containing protease inhibitor PMSF was added to each well. The cells were scraped off with a scraper and lysed on ice for 30 min and then collected into a 1.5 mL centrifuge tube, centrifuged for 15 min at 4 °C, the supernatant was aspirated and stored at −80 °C for storage to measure concentration. The nuclear and cytoplasmic proteins were extracted by Nuclear and Cytoplasmic Protein Extraction Kit (KGP150/KGP1100, KeyGEN BioTECH, Nanjing, Jiangsu, China).

### 4.7. The Detection of Tissue and Cellular Protein Concentration 

Protein concentration was detected by The Biyuntian BCA test kit. The procedure was as follows: Prepared a 100 μL system standard curve according to the BCA specification; prepared BCA working solution, 20 μL per sample and BCA working solution 180 μL was added into a 96-well microplate; mixed and incubated at 37 °C for 30 min. Then measured the absorbance at 562 nm with a microplate reader; calculated the protein concentration according to the standard curve.

### 4.8. Western Blot Analysis

The sample was added to the electrophoresis well of the pre-formed gel and electrophoresed (The concentrated glue voltage: 60 V, 30 min; the separation glue: 90 V, 120 min). Then placed the separation rubber on the filter paper, covered the film on the glue, and placed three sheets of filter paper on the film. Finally, covered the sponge pad. Combined the sandwich clips and insert them into the transfer slot. Turned on the power (90 V constant pressure); Placed the transferred membrane on a decolorizing shaker at room temperature, add 5% skim milk (0.5% TBST) and blocked for 1 h; Dilute the primary concentrated solution with 5% skim milk (0.5% TBST) to the appropriate concentration and incubate overnight at 4 °C; The membrane was flushed three times with a configured TBST on a decolorizing shaker at room temperature for 10 min each time. The secondary antibody was diluted 5000 times with TBST, incubated for 60 min at room temperature, and washed three times with TBST on a bleaching shaker 5 min each time. The two reagents of ECL A and ECL B were mixed with a medium volume in a centrifuge tube, and the protein side of the PVDF membrane was brought into contact with the mixed solution in the upward direction. Removed the residual liquid and exposed in an exposure apparatus after 1 min. The Image J software processing system was used to analyze the gray value of the target strip. The antibodies used in this study include GAPDH (CST, Boston, MA, USA), Tublin (CST, Boston, MA, USA), Bax (Proteintech Co LTD, Wuhan, Hubei, China), BCL2 (Proteintech Co LTD, Wuhan, Hubei, China), HSP90 (Proteintech Co LTD, Wuhan, Hubei, China), HSP70 (Proteintech Co LTD, Wuhan, Hubei, China), GRP78 (Proteintech Co LTD, Wuhan, Hubei, China), CHOP (CST, Boston, MA, USA), HSF1(CST, Boston, MA), CDK5RAP3 (Proteintech Co LTD, Wuhan, Hubei, China).

### 4.9. Trimerization Detection Experiment

The cells were removed from the CO_2_ incubator with the medium removed, washed 3 times with PBS, and the cells were lysed with IP lysate (containing protease inhibitors PMSF and PI), and scraped off with a cell scraper and transferred to a 1.5 mL EP tube. The ice was cleaved for 15 min with constant shaking to fully lysis. Transferring the supernatant to a new EP tube after centrifugation at rpm for 10 min at 4 °C; adding freshly prepared 2.3% glutaraldehyde (5 μL per 100 μL IP lysate) to a final concentration of 0.12%. After cross-linking for 5 min at room temperature, it was terminated with adding 1M Tris-HCl (pH 8.0) (Tris-HCl: glutaraldehyde = 2:1); the sample was boiled by adding 5 × Loading Buffer, then frozen and stored in a refrigerator at −20 °C and detected by Western Blot.

### 4.10. Co-Immunoprecipitation

Immunoprecipitation assays bovine mammary tissue [50]. The lysates were pre-cleared with protein A+G agarose beads (Beyotime Biotechnology, P2012), and the supernatant was incubated overnight with an anti-HSF1 or anti-CDK5RAP3 antibody on a rotating platform at 4 °C and then incubated with protein A+G agarose beads. The beads were collected, washed, and resuspended in equal volumes of 5 SDS loading buffer. The immunoprecipitation fractions were separated using 8% SDS-PAGE. A western blotting assay was performed as described.

### 4.11. Immunofluorescent Staining

Cell immunofluorescence staining was conducted after fixing cells with 4% paraformaldehyde for 30 min and permeabilized with 1% Triton X-100. 5% BSA was utilized to block the non-specific binding for 30 min at room temperature. Specimens were later incubated in primary antibodies (CDK5RAP3, Abnova, Taiwan, China; HSF1, CST, Boston, MA, USA) in the presence of 5% BSA at 4 °C overnight. The sections were washed in PBST and followed by incubation with the secondary antibodies including Fluorescein-conjugated Goat Anti-Mouse IgG (H+L) (1:100) (ZSGB-BI, Beijing, China) and Rhodamine (TRITC)-Conjugated Goat Anti-Rabbit IgG (H+L) (1:100) (ZSGB-BIO, Beijing, China) at 37 °C for 1 h. The sections were then counterstained with 4, 6 diamidino-2 phenyls-indole (DAPI; Sigma-Aldrich, St. Louis, MO, USA) for nuclear staining. Images were taken by a confocal microscope (Carl Zeiss 700, Göttingen, Germany).

### 4.12. Statistical Method

Statistical analysis was conducted through analysis of variance (ANOVA) and Student’s *t*-test, using GraphPad Prism7 software. The data shown are representative of at least three independent experiments. The *p*-value of *p* < 0.05 was considered to indicate a statistically significant difference. The *p* < 0.001 was considered to be an extremely significant difference.

## Figures and Tables

**Table 1:** The primers used for qRT-PCR analysis.

Gene	Primer Sequences
mβ-ACTIN	Forward: 5′-GACCTCTATGCCAACACAGT-3′
	Reverse: 5′-AGTACTTGCGCTCAGGAGGA-3′
mGAPDH	Forward: 5′-AACTTTGGCATTGTGGAAGG-3′
	Reverse: 5′-ACACATTGGGGGTAGGAACA-3′
mCDK5RAP3	Forward: 5′-ATGAGATCGACTGGGGTGAC-3′
	Reverse: 5′-AGCCTCAGTTCCTGTCTCCA-3′
mHSF1	Forward: 5′-TCATCTGCTGGAGCCCGAGTG-3′
	Reverse: 5′-ATGTTGAGCTGCCGCACGAAG-3′
bβ-ACTIN	Forward: 5′-GGGCAGGTCATCACCATCGG-3′
	Reverse: 5′-TCATTGTGCTGGGTGCCAGG-3′
bGAPDH	Forward: 5′-AAGGTCGGAGTGAAC-3′
	Reverse: 5′-CGTTCTCTGCCTTGACTGTG-3′
bCDK5RAP3	Forward: 5′-TCGACTGGCTGGTAGACAGAAGG-3′
	Reverse: 5′-GTCCTGGATGGCGGCATTGATC-3′
bHSP90	Forward: 5′-CCAAGTCTGGCACTAAAG-3′
	Reverse: 5′-GAAGACTCCCAAGCATAC-3′
bHSF1	Forward: 5′-AAAGATCCCCCTGATGCTGAACGAC-3′
	Reverse: 5′-CAGTTCGGTGATGTCGGAGATGATG-3′

m: mouse; b: bovine.

## References

[1] Sanh M.V., Wiktorsson H., Ly L.V. (2007). Effect of feeding level on milk production, body weight change, feed conversion and postpartum oestrus of crossbred lactating cows in tropical conditions. J. Shanxi Coll. Tradit. Chin. Med..

[2] Wang J., Xue X., Liu Q., Zhang S., Peng M., Zhou J., Chen L., Fang F. (2019). Effects of duration of thermal stress on growth performance, serum oxidative stress indices, the expression and localization of ABCG2 and mitochondria ROS production of skeletal muscle, small intestine and immune organs in broilers. J. Biol..

[3] Bhanuprakash V., Singh U., Sengar G., Sajjanar B., Bhusan B., Raja T.V., Alex R., Kumar S., Singh R., Ashish K. (2016). Differential effect of thermal stress on HSP70 expression, nitric oxide production and cell proliferation among native and crossbred dairy cattle. J. Biol..

[4] Badri T.M., Chen K.L., Alsiddig M.A., Li L., Cai Y., Wang G.L. (2018). Genetic polymorphism in Hsp90AA1 gene is associated with the thermotolerance in Chinese Holstein cows. Cell Stress Chaperones.

[5] Trifkovic J., Jovanovic L., Duric M., Stevanovic-Dordevic S., Milanovic S., Lazarevic M., Sladojevic Z., Kirovski D. (2018). Influence of different seasons during late gestation on Holstein cows’ colostrum and postnatal adaptive capability of their calves. Int. J. Biometeorol..

[6] Polsky L., von Keyserlingk M.A.G. (2017). Invited review: Effects of heat stress on dairy cattle welfare. J. Dairy Sci..

[7] Song M., Yang X., Ren X., Maliskova L., Li B., Jones I.R., Wang C., Jacob F., Wu K., Traglia M. (2019). Mapping cis-regulatory chromatin contacts in neural cells links neuropsychiatric disorder risk variants to target genes. Nat. Genet..

[8] Almeida R.A., Kerro-Dego O., Rius A.G. (2018). Effect of heat stress on the interaction of Streptococcus uberis with bovine mammary epithelial cells. J. Dairy Res..

[9] Takii R., Fujimoto M., Matsumoto M., Srivastava P., Katiyar A., Nakayama K.I., Nakai A. (2019). The pericentromeric protein shugoshin 2 cooperates with HSF1 in heat shock response and RNA Pol II recruitment. EMBO J..

[10] Pincus D. (2020). Regulation of Hsf1 and the Heat Shock Response. Adv. Exp. Med. Biol..

[11] Yin C.F., Kao S.C., Hsu C.L., Chang Y.W., Cheung C.H.Y., Huang H.C., Juan H.F. (2020). Phosphoproteome Analysis Reveals Dynamic Heat Shock Protein 27 Phosphorylation in Tanshinone IIA-Induced Cell Death. J. Proteome Res..

[12] Workman P. (2020). Reflections and Outlook on Targeting HSP90, HSP70 and HSF1 in Cancer: A Personal Perspective. Adv. Exp. Med. Biol..

[13] Bhatti M., Dinn S., Miskiewicz E.I., MacPhee D.J. (2019). Expression of heat shock factor 1, heat shock protein 90 and associated signaling proteins in pregnant rat myometrium: Implications for myometrial proliferation. Reprod. Biol..

[14] Bickel D., Gohlke H. (2019). C-terminal modulators of heat shock protein of 90kDa (HSP90): State of development and modes of action. Bioorg. Med. Chem..

[15] Gaur D., Singh P., Guleria J., Gupta A., Kaur S., Sharma D. (2020). The Yeast Hsp70 Co-chaperone Ydj1 Regulates Functional Distinction of Ssa Hsp70s in the Hsp90 Chaperoning Pathway. Genetics.

[16] Gomez-Pastor R., Burchfiel E.T., Thiele D.J. (2018). Regulation of heat shock transcription factors and their roles in physiology and disease. Nat. Rev. Mol. Cell Biol..

[17] Katiyar A., Fujimoto M., Tan K., Kurashima A., Srivastava P., Okada M., Takii R., Nakai A. (2020). HSF1 is required for induction of mitochondrial chaperones during the mitochondrial unfolded protein response. FEBS Open Bio..

[18] Yang R., Wang H., Kang B., Chen B., Shi Y., Yang S., Sun L., Liu Y., Xiao W., Zhang T. (2019). CDK5RAP3, a UFL1 substrate adaptor, is crucial for liver development. Development.

[19] Jiang H., Luo S., Li H. (2005). Cdk5 activator-binding protein C53 regulates apoptosis induced by genotoxic stress via modulating the G2/M DNA damage checkpoint. J. Biol. Chem..

[20] Wang X., Ching Y.P., Lam W.H., Qi Z., Zhang M., Wang J.H. (2000). Identification of a common protein association region in the neuronal Cdk5 activator. J. Biol. Chem..

[21] Chen Q.Y., Liu L.C., Wang J.B., Xie J.W., Lin J.X., Lu J., Cao L.L., Lin M., Tu R.H., Huang C.M. (2019). CDK5RAP3 Inhibits the Translocation of MCM6 to Influence the Prognosis in Gastric Cancer. J. Cancer.

[22] Wang J.B., Wang Z.W., Li Y., Huang C.Q., Zheng C.H., Li P., Xie J.W., Lin J.X., Lu J., Chen Q.Y. (2017). CDK5RAP3 acts as a tumor suppressor in gastric cancer through inhibition of beta-catenin signaling. Cancer Lett..

[23] Chen J., Shi Y., Li Z., Yu H., Han Y., Wang X., Sun K., Yang T., Lou K., Song Y. (2011). A functional variant of IC53 correlates with the late onset of colorectal cancer. Mol. Med..

[24] Mak G.W., Chan M.M., Leong V.Y., Lee J.M., Yau T.O., Ng I.O., Ching Y.P. (2011). Overexpression of a novel activator of PAK4, the CDK5 kinase-associated protein CDK5RAP3, promotes hepatocellular carcinoma metastasis. Cancer Res..

[25] Stav D., Bar I., Sandbank J. (2007). Usefulness of CDK5RAP3, CCNB2, and RAGE genes for the diagnosis of lung adenocarcinoma. Int. J. Biol. Markers.

[26] Wamsley J.J., Gary C., Biktasova A., Hajek M., Bellinger G., Virk R., Issaeva N., Yarbrough W.G. (2017). Loss of LZAP inactivates p53 and regulates sensitivity of cells to DNA damage in a p53-dependent manner. Oncogenesis.

[27] Jiang H., Wu J., He C., Yang W., Li H. (2009). Tumor suppressor protein C53 antagonizes checkpoint kinases to promote cyclin-dependent kinase 1 activation. Cell Res..

[28] Cai Y., Pi W., Sivaprakasam S., Zhu X., Zhang M., Chen J., Makala L., Lu C., Wu J., Teng Y. (2015). UFBP1, a Key Component of the Ufm1 Conjugation System, Is Essential for Ufmylation-Mediated Regulation of Erythroid Development. PLoS Genet..

[29] Lin J.X., Xie X.S., Weng X.F., Zheng C.H., Xie J.W., Wang J.B., Lu J., Chen Q.Y., Cao L.L., Lin M. (2018). Low expression of CDK5RAP3 and DDRGK1 indicates a poor prognosis in patients with gastric cancer. World J. Gastroenterol..

[30] Zheng C.H., Wang J.B., Lin M.Q., Zhang P.Y., Liu L.C., Lin J.X., Lu J., Chen Q.Y., Cao L.L., Lin M. (2018). CDK5RAP3 suppresses Wnt/beta-catenin signaling by inhibiting AKT phosphorylation in gastric cancer. J. Exp. Clin. Cancer Res..

[31] Lin J.X., Weng X.F., Xie X.S., Lian N.Z., Qiu S.L., Wang J.B., Lu J., Chen Q.Y., Cao L.L., Lin M. (2019). CDK5RAP3 inhibits angiogenesis in gastric neuroendocrine carcinoma by modulating AKT/HIF-1alpha/VEGFA signaling. Cancer Cell Int..

[32] Egusquiaguirre S.P., Liu S., Tosic I., Jiang K., Walker S.R., Nicolais M., Saw T.Y., Xiang M., Bartel K., Nelson E.A. (2020). CDK5RAP3 is a co-factor for the oncogenic transcription factor STAT3. Neoplasia.

[33] Wang J., An H., Mayo M.W., Baldwin A.S., Yarbrough W.G. (2007). LZAP, a putative tumor suppressor, selectively inhibits NF-kappaB. Cancer Cell.

[34] Zou Y., Shao J., Li Y., Zhao F.Q., Liu J.X., Liu H. (2019). Protective Effects of Inorganic and Organic Selenium on Heat Stress in Bovine Mammary Epithelial Cells. Oxid. Med. Cell Longev..

[35] Cai M., Hu Y., Zheng T., He H., Xiao W., Liu B., Shi Y., Jia X., Chen S., Wang J. (2018). MicroRNA-216b inhibits heat stress-induced cell apoptosis by targeting Fas in bovine mammary epithelial cells. Cell Stress Chaperones.

[36] Xiao Y., Rungruang S., Hall L.W., Collier J.L., Dunshea F.R., Collier R.J. (2017). Effects of niacin and betaine on bovine mammary and uterine cells exposed to thermal shock in vitro. J. Dairy Sci..

[37] Xu J., Yin B., Huang B., Tang S., Zhang X., Sun J., Bao E. (2019). Co-enzyme Q10 protects chicken hearts from in vivo heat stress via inducing HSF1 binding activity and Hsp70 expression. Poult. Sci..

[38] Calderwood S.K., Gong J., Murshid A. (2016). Extracellular HSPs: The Complicated Roles of Extracellular HSPs in Immunity. Front. Immunol..

[39] Wu J., Liu T., Rios Z., Mei Q., Lin X., Cao S. (2017). Heat Shock Proteins and Cancer. Trends Pharm. Sci..

[40] Bepperling A., Alte F., Kriehuber T., Braun N., Weinkauf S., Groll M., Haslbeck M., Buchner J. (2012). Alternative bacterial two-component small heat shock protein systems. Proc. Natl. Acad. Sci. USA.

[41] Kampinga H.H., Hageman J., Vos M.J., Kubota H., Tanguay R.M., Bruford E.A., Cheetham M.E., Chen B., Hightower L.E. (2009). Guidelines for the nomenclature of the human heat shock proteins. Cell Stress Chaperones.

[42] Erlejman A.G., Lagadari M., Toneatto J., Piwien-Pilipuk G., Galigniana M.D. (2014). Regulatory role of the 90-kDa-heat-shock protein (Hsp90) and associated factors on gene expression. Biochim. Biophys. Acta.

[43] Raychaudhuri S., Loew C., Korner R., Pinkert S., Theis M., Hayer-Hartl M., Buchholz F., Hartl F.U. (2014). Interplay of acetyltransferase EP300 and the proteasome system in regulating heat shock transcription factor 1. Cell.

[44] Budzynski M.A., Puustinen M.C., Joutsen J., Sistonen L. (2015). Uncoupling Stress-Inducible Phosphorylation of Heat Shock Factor 1 from Its Activation. Mol. Cell Biol..

[45] Millson S.H., Piper P.W. (2014). Insights from yeast into whether the inhibition of heat shock transcription factor (Hsf1) by rapamycin can prevent the Hsf1 activation that results from treatment with an Hsp90 inhibitor. Oncotarget.

[46] Tang Z., Dai S., He Y., Doty R.A., Shultz L.D., Sampson S.B., Dai C. (2015). MEK guards proteome stability and inhibits tumor-suppressive amyloidogenesis via HSF1. Cell.

[47] Sharma P., Sharma A., Sodhi M., Verma P., Parvesh K., Swami S.K., Jast A., Shandilya U.K., Mukesh M. (2019). Characterizing binding sites of heat responsive microRNAs and their expression pattern in heat stressed PBMCs of native cattle, exotic cattle and riverine buffaloes. Mol. Biol. Rep..

[48] Roth Z. (2017). Effect of Heat Stress on Reproduction in Dairy Cows: Insights into the Cellular and Molecular Responses of the Oocyte. Annu. Rev. Anim. Biosci..

[49] Zhang M., Zhu X., Zhang Y., Cai Y., Chen J., Sivaprakasam S., Gurav A., Pi W., Makala L., Wu J. (2015). RCAD/Ufl1, a Ufm1 E3 ligase, is essential for hematopoietic stem cell function and murine hematopoiesis. Cell Death Differ..

[50] Zhang X., Yang Y., Xia Q., Song H., Wei R., Wang J., Zou K. (2018). Cadherin 22 participates in the self-renewal of mouse female germ line stem cells via interaction with JAK2 and beta-catenin. Cell Mol. Life Sci..

